# Case Study and Qualitative Analysis of Emergency Department Community Advisory Council on Intimate Partner Violence

**DOI:** 10.5811/westjem.47456

**Published:** 2025-12-23

**Authors:** Eva Kitlen, Alice Lu, Katrin Jaradeh, Stephanie Lawless, Elizabeth Raby, Theresa Cheng, Leigh Kimberg, Christopher R. Peabody

**Affiliations:** * University of California, San Francisco School of Medicine, San Francisco, California; † University of California, San Francisco, San Francisco General Hospital, Department of Emergency Medicine, San Francisco, California; ‡ University of California, San Francisco, San Francisco General Hospital, Division of General Internal Medicine, San Francisco, California; § Mass General Brigham, Department of Emergency Medicine, Boston, Massachusetts

## Abstract

**Introduction:**

As part of a quality improvement initiative, our emergency department (ED) implemented a community advisory council consisting of leaders from five community-based organizations (CBO) that provide services for survivors of intimate partner violence. We used qualitative interviews with participants from the organizations to evaluate the council by identifying factors that promoted and hindered their engagement in this partnership between the community and the ED as well as best practices for future collaborations

**Methods:**

We conducted five, 30-minute semi-structured interviews, one for each CBO representative on the council. Interview questions were based on validated toolkits for evaluating community-based participatory research. We conducted thematic analysis using a barriers and facilitators framework.

**Results:**

Our focus on building relationships within the community advisory council facilitated collaboration between the ED and the CBOs. We identified structural barriers to and facilitators of the relationship-building process, as well as four behaviors that promoted relationship-building within the council. These behaviors included a joint problem-solving orientation, a culture of curiosity, shared empathy between emergency clinicians and CBO members, and a deeper understanding of barriers to caring for survivors of intimate partner violence in the ED. Themes regarding the impact of the council included the results of tangible projects as well as cultural shifts in the ED as perceived by leaders of the CBOs.

**Conclusion:**

We share a case study of a collaboration between the ED and community-based organizations that illustrates barriers to and facilitators of engagement by leaders of these organizations in community-healthcare partnerships. The ED is a short but meaningful stop in recovery for many survivors, and a warm handoff to a CBO can be an essential next step in their care. When rooted in mutually respectful, trusting relationships, ED-CBO partnerships have the potential to enable survivor-centered, quality improvement efforts that work to improve the continuum of care between the ED and the community.

## INTRODUCTION

### Background

Intimate partner violence is a public health concern with high prevalence in the emergency department (ED). Approximately 23% of partnered patients presenting to EDs in urban, safety-net hospitals have experienced intimate partner violence.^1^–^3^ At our hospital, an urban Level I trauma center and safety-net hospital, over 400 patients per year report current intimate partner violence during ED triage screening. This number underestimates the prevalence as it does not capture survivors who disclose later or those who are not comfortable disclosing. Previously, the American College of Emergency Physicians called for EDs to partner with community-based organizations (CBO) to provide care for survivors of intimate partner violence.^4^ Immediate referrals from the ED to CBOs increase the likelihood that survivors engage with support services,^5^ especially when these referrals include direct handoffs to CBOs.^6^–^8^

In 2023, as part of a quality improvement (QI) initiative using a human-centered design framework^9^ regarding ED care of survivors of intimate partner violence, our ED implemented a community advisory council consisting of leaders from five local CBOs that provide culturally relevant services for these survivors, including emergency hotlines, accompaniment when seeking medical or legal care, emergency and long-term housing, and counseling. This group partnered with ED staff to form a community-healthcare partnership. The goal of forming this partnership with CBO leaders was to seek their input on how ED staff could more effectively to connect survivors to their resources in the community. Through this partnership, CBO leaders acted as consultants for a QI initiative focused on intimate partner violence and ED-based patient education.

In its first year, the community-healthcare partnership conducted four meetings that included the community advisory council, emergency physicians, social workers, physician-leaders in intimate partner violence advocacy, and medical students to solicit input on developing survivor-centered care pathways in the ED (Appendix 1). Meetings involved discussions of survivors’ experiences in the ED, a gap analysis of ED practices, a tour of the ED, and discussion of a resident training curriculum and patient education materials.^10^,^11^

### Objectives

After the first year of community-healthcare partnership meetings, we evaluated the community advisory council by using qualitative interviews with council members to explore the barriers to and the facilitators of CBO participation in the partnership. We sought to understand the CBO perspective on our collaboration, particularly with regard to providing meaningful connections to community resources for survivors seeking care in the ED.

## METHODS

### Setting

The community advisory council was formed according to our university’s Center for Community Engagement guidelines. We compensated participants for their time at the university’s suggested hourly rate.^12^ We had funding for one year of quarterly meetings. To reflect the diversity of our patient population and the disproportionate impact of intimate partner violence in marginalized communities, we recruited representatives from CBOs that provide culturally relevant and identity-concordant care for survivors.

**Population Health Research Capsule**

What do we already know about this issue?

*Survivors of intimate partner violence seen in the emergency department (ED) are more likely to engage with support services if the ED immediately refers them to community-based services*.

What was the research question?

*What factors encourage engagement by a community-based organization (CBO) in an ED-based community-healthcare partnership?*

What was the major finding of the study?

*This qualitative study identified relationship-building as central to effective ED-CBO collaboration*.

How does this improve population health?

*This study offers guidance for EDs seeking to collaborate with CBOs to improve their care of survivors of intimate partner violence*.

### Study Design

Interviewers used a semi-structured interview guide developed by authors EK and CP based on validated toolkits for evaluating community-based participatory research projects, which includes a guide from the University of New Mexico and community engagement guidelines from the US Centers for Disease Control and Prevention (Appendix 2).^13^–^16^ All participants provided verbal consent to participate in the study. No identifying information was collected. Participants received a $25 gift card in lieu of the hourly rate. Our organization’s institutional review board determined this study was exempt (IRB #21-35510). Interviews lasted 30 minutes each and were conducted from June-October 2024. We interviewed all available participants, ie, the five CBO leaders (one from each organization).

### Measurements and Analysis

Author EK conducted all interviews; SL co-conducted one interview. Interviews were conducted via videoconferencing, and they were recorded and transcribed using Zoom teleconferencing software v6.0.11 (Zoom Communications, San Jose, CA). Authors EK, CP, and LK reviewed the transcripts for accuracy. Recordings and transcripts were stored within our institution’s secure, cloud-based file collaboration software.

Authors EK and KJ coded the interview transcripts using Atlas.ti v24 software for qualitative analysis (Lumivero, LLC, Berlin, Germany). Our coding methods focused on enhancing credibility, dependability, and confirmability as per validated trustworthiness frameworks for establishing rigor in qualitative research.^17^ Following the code-recode strategy, we used two coders. EK was an “inside” coder, having participated in two of four community-healthcare partnership meetings; KJ was an “outside” coder who did not participate in the meetings. We applied a barriers and facilitators framework to understand participants’ experiences with the partnership. Following the constant comparison approach, EK and KJ reviewed and open-coded one transcript at a time; they then met to refine the code book. Author CP independently reviewed the codes and met with EK to construct the codes into higher order themes. We kept a detailed audit trail in the code book. To allow for peer debriefing, the full investigator team met multiple times to discuss patterns and resolve any discrepancies.^18^,^19^

## RESULTS

We identified thematic barriers to and facilitators of ED-CBO collaboration in a QI setting, illustrated in the Table and the Figure. The primary theme was relationship-building as central to the formation, operation, and impact of a community-healthcare partnership.We organized sub-themes as follows: structural barriers, structural facilitators, behavioral facilitators of relationship-building, and the impact of these relationships.

### Structural Facilitators of Relationship Building

Long-standing relationships between CBO leaders and physician-leaders in intimate partner violence advocacy facilitated the formation of the community advisory council and the community-healthcare partnership. Participants stated that they were more willing to join the partnership because they were invited by someone they trusted. Likewise, pre-existing relationships between the CBOs made it easier to begin collaborative work in the council.

Other structural facilitators included psychological safety, human-centered design exercises, and an in-person tour of the ED for the community advisory council. Psychological safety emerged from trust, clear communication, and prioritizing CBO perspectives. Human-centered design resonated with CBO leaders because of its similarities with trauma-informed care. Both approaches center empathy, trust, and autonomy, which connected the operations of the community-healthcare partnership with CBO practices. The ED tour built appreciation for ED staff and an understanding of ED resource limitations. Setting realistic goals and making reasonable requests of CBO representatives facilitated the council’s impact and productivity.

### Structural Barriers to Relationship-building

Participants in the community advisory council described that some of their experiences with prior healthcare partnerships were a barrier to relationship-building in the community-healthcare partnership, as prior partners had sought CBO support without centering their feedback. Interruptions to relationship-building, including incomplete attendance at meetings due to scheduling challenges and a lack of personnel continuity within the partnership, impeded the process of strengthening connections.

### Behavioral Facilitators of Relationship-building

We identified four sub-themes representing key behaviors that allowed for relationship-building in the community-healthcare partnership. First, CBOs and healthcare staff shared a **joint problem-solving orientation**—the understanding that the ED and CBOs share similar problems when caring for survivors of intimate partner violence and that collaboration is necessary to solve these problems. A culture of curiosity fostered a sense of partnership, referring to an environment where healthcare staff valued CBO perspectives and were open to discussing difficulties without needing an immediate solution, which was facilitated by psychological safety.

Using human-centered design exercises in the community-healthcare partnership meetings^20^ helped build empathy between the council and the ED team. Placing advisory council members and their lived experiences at the heart of the problem-solving process allowed team members to better understand each other’s work. Ultimately, these behaviors created a deeper shared understanding of the barriers to care for survivors of intimate partner violence in the ED. The in-person tour of the ED was the key to facilitating this understanding.

### Impact of Relationship-building

Forming relationships through the community-healthcare partnership led to tangible outcomes including a digital tool to help clinicians navigate resources,^21^ an intimate partner violence training curriculum for ED residents, and a second year of funding for the advisory council. They also improved trust: CBO leaders felt more comfortable interfacing with the ED after participating in the community advisory council because they knew that ED staff were aware of intimate partner violence and survivors’ needs.

## DISCUSSION

Our findings suggest that the effectiveness of community advisory councils in QI efforts in the ED. depends not only on their composition but also on how relationships are built and sustained. The council became more than a consultative body: It evolved into a collaborative space where community and ED stakeholders could co-create solutions grounded in empathy, trust, and cooperation. Structural facilitators enabled participation, while behavioral facilitators fostered alignment across different forms of expertise. These elements improved trust between the ED teams and CBO leaders, supporting critical connections between ED teams caring for survivors of intimate partner violence and CBO leaders who could offer long-term support.

Ultimately, we identified this foundation of trust as the key to our partnership. This is consistent with research on ED teamwork that establishes mutual trust and respect as a central tenet of effective ED teamwork.^22^ Future research should explore the roles of CBO members in ED teams and frameworks for understanding ED-CBO partnerships in the broader context of ED teamwork. This trust and teamwork also led to deliverables that inspired further work in this field, including our digital resource navigation tool^21^ and a two-part, trauma-informed care curriculum for ED residents. We can build on the general understanding of ED-CBO partnerships outlined here by studying what makes these partnerships successful in the context of a discrete project.

## LIMITATIONS

Our small sample size and location at a single site limit the external validity of our findings. Interviewer reflexivity is a consideration as the primary interviewer participated in meetings of the community-healthcare partnership. Additionally, while the financial compensation ($25 gift card) may have influenced what participants felt comfortable sharing, it was in line with standard practice at our institution.

As described in the “Methods” section of this paper, we used the trustworthiness framework to enhance internal and external validity, dependability, and confirmability.^17^ According to the principle of prolonged engagement, the longevity of our work with the CBOs supports our confidence in our findings and, thus, our internal validity. We also used triangulation (multiple authors reviewing the codebook) and peer debriefing to increase internal validity. We used thick description to enhance external validity by providing detailed contextual information about our study that will allow others to determine what aspects of our findings apply to their settings. Our coding and analysis methods, including the code-recode strategy, an audit trail, and reflecting on reflexivity, enhanced dependability and confirmability.

## CONCLUSION

We present a case study that offers guidance for EDs seeking to partner with community-based organizations. When grounded in trust and mutual respect, these partnerships have the potential to strengthen the continuum of care, enabling survivor-centered quality improvement efforts that begin in the ED and extend into the community organizations that patients engage with after discharge.

## Supplementary Information





## Figures and Tables

**Figure 1:**
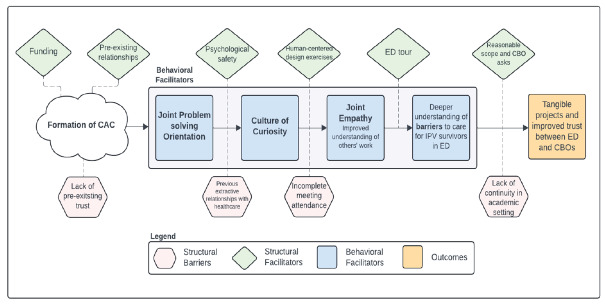
Flowchart illustrating barriers to and facilitators of collaboration between an emergency department and community-based organizations in the setting of a community advisory council focused on care for survivors of intimate partner violence. Green trapezoids show structural facilitators, pink hexagons show structural barriers, blue squares show behavioral facilitators, and the orange square represents outcomes. *CAC*, community advisory council; *CBO*, community-based organizations; *ED*, emergency department; *IPV*, intimate partner violence.

**Table:** Facilitators of and barriers to a collaboration between the emergency department and community-based organizations in the setting of a community advisory council.

Overarching facilitator	
Focus on relationships	*“I think pretty much all the time, relationships, like direct personal relationships, are what make anything work. So, no matter what, just the fact of having the partnership and meeting a couple of people just means that…any thickness that gets added into the relationship will help.” [2]* *“I’m happy…to have other places or people that I could reach out to…it’s all about making connections, right? …that’s huge, just [that] you can have a conversation with someone [in the ED].” [3]* *“I felt like it was a drive to, I don’t know how to describe it…like deliverables, deliverables, deliverables, and it was more like I wanted the human aspect…” [5]*
Structural facilitators	
Pre-existing relationships with healthcare teams	*“I was happy to participate. I mean, there’s that relationship and trust built already with Dr ... So I was like, of course, of course, we’ll do it.” [1]*
Pre-existing relationships between CBOs	*“There’s something to...having your long-term collaborators that you’re working with really steadily and closely and then you go into a new space with them”… [1]*
Psychological safety	*“...just … being in [the ED] I was like, ‘Wow, I don’t know. I really don’t know what to tell you.’ And...my concern wasn’t shut down at all. You know, the people who were present were very like, ‘Okay, let’s talk about that.’ You know it, it was a very open space of ‘Share your thoughts. That’s why you’re here. That’s why we brought you here.’ So there was no judgment. It was just listening. And it seems like a sincere like, ‘I actually sincerely want to know what you’re thinking about this.’” [1]*
Human-centered design exercises	***“*** *Our day-to-day practice, since we opened, is so focused on creating that [human-centered approach]…I could tell that there was a similar brand of conscientiousness in designing the conversation.” [2]*
Emergency department tour	*“I did the tour in the emergency room and it was...having us...give our feedback. So like, okay, they really care what we think, how we can make it better, how they can make it better.” [4]*
Reasonable scope and asks for CBOs	*“I think the scope of what was proposed was reasonable…it’s so nice when it’s clear that somebody is going to be…responsible for driving it. And they’re going to ask you about every part of the journey. So you’re like a member of the car, you know. But you’re not steering the car.” [2]*
Structural barriers	
Absence of prior trust between EDs and CBOs	*”I think in general, people…feel like it’s just complete luck of the draw when you go into a[n] institution; it’s like, who you’re going to get and what’s going to happen to you is completely out of your control.‬ And that you cannot count on continuity…It’s just scary. So, I think just, you know, having any relationship always makes people feel a teeny bit more like, maybe everything isn’t an unknown.” [2]*
Previous extractive relationships with healthcare	*“I’ve definitely been a part of other committees where that wasn’t present, where…it was kind of clear, it’s like what would be helpful would be your buy-in. And how do we make this work versus oh, no, we’re actually open to hearing that this…this is hard. And you don’t need to have solutions, you know.” [1]*
Incomplete meeting attendance	*“…the conversation…in May of 2023...had so many people there…the important perspectives needed to be captured, and when you don’t have all of that there, things can get set up, and they are not taken into consideration how it’s going to affect ABCDE. Right? You only have parts of the spokes there, and you have no ability to know how it’s going to affect the rest of the spokes.” [3]*
Lack of personnel continuity in an academic setting	***“*** *…because there’s a school component, there’s always going to be rotation of people. So that just means that to make it sustainable…the succession, to make sure that somebody is there enough that they can pick it up without it falling down will be important.” [2]*
Behavioral facilitators	
Joint problem-solving orientation	*“Coming together and having conversations and learning about what each other is, you know, doing and confronted with and where the challenges are, and how we might be able to work together to kind of answer some of those, or just learn, it’s nice. I think there’s real value in breaking out of your little silos.” [1]*
Culture of curiosity	*“I did feel there was a sense of real curiosity on behalf of the committee to kind of get our community, the DV community’s, input and kind of like just opinions and thoughts on what was going on, and assembling us together, and then actually having, like a culture of curiosity, I think, came through really nicely.” [1]*
Joint empathy—shared understanding of each other’s work	*“So [touring the ED] builds like an allyship, and again, just more kind of empathy…I remember those days, like at the shelter, people coming in...you don’t know what’s going to happen any given day. It’s hard. It’s hard to navigate. So having that experience, I think, gives us a better idea of how we might be able to work together.” [1]*
Deeper understanding of barriers to care for IPV survivors in the ED	*“I’m very visual. So like I was imagining like when we walk [through the ED], and they have to sit in there thinking that they might even be with their abuser sitting in there like, how can we know…how we can make it so they can be in a different room.” [4]* *“People are a lot more aware of, it’s not just about the medicine, but also starting to think about…where are we talking to [survivors]? How might somebody experience coming into that ER?*

*CBO*, community-based organization; *DV*, domestic violence; *ED*, emergency department; *IPV*, intimate partner violence.
