# Supplementary material for: Case Study and Qualitative Analysis of Emergency Department Community Advisory Council on Intimate Partner Violence
Source: West J Emerg Med. 2025 Dec 23;27(1):114–20. doi: 10.5811/westjem.47456 (PMC12815508; doi:10.5811/westjem.47456)
Supplement: Supplementary file 1 [file wjem-27-114-s001.docx]

APPENDIX 1: Meeting Structure

| **Meeting** | **Dates & Location** | **Participants** | **Objectives & Activities** | **Gaps Identified** |
| --- | --- | --- | --- | --- |
| 1 | 5/15/2023, Zoom | 5/5 CBO representatives  2 ED attending physicians  DPH IPV physician lead  ED social worker  Medical student coordinators | **Defining the problem:** Identify ideal state of practice and gaps between that state and current practice using human-centered design exercises | ED-CBO communication regarding general practices and care for individual survivors  Culturally relevant resources  Projects focused on directly addressing inequities in care  Referral and communication logistics |
| 2 | 9/25/2023, Zoom | 5/5 CBO representatives, 2 additional CBO leaders  DPH IPV physician lead  ED social worker  Medical student coordinators | **Workshopping interventions**: Discuss ideas from meeting 1 regarding training in the ED and institutional policies; better understand benefits and challenges of a training-focused intervention | Limited CBO capacity, especially in certain months  Need to center CBO perspectives in training  Lack of current knowledge of training programs and ED residents’ knowledge |
| 3 | 1/26/2024, 3/6/2024, 3/11/2024,  In-person | 2/5 CBO representatives on 1/26, 1/5 on 3/6, and 2/5 on 3/11  1 ED attending physician  DPH IPV physician lead  ED social worker  Medical student coordinators | **Tour of the ED:** Walk through the path a survivor would take in the ED together to better understand gaps in care and barriers to care; use this to inform feedback on the Discharge Navigator IPV module | Lack of confidential spaces for IPV screening in ED  Challenges teams face given busy ED environment  Need for universal trauma-informed care in the ED  Challenges with maintaining accuracy of Discharge Navigator module  Structural racism built into Discharge Navigator module: Black/African American left out of “identity” filter based on lack of acute resources in the local community |
| 4 | 5/13/2024, 6/12/2024, 7/18/2024,  Zoom | 3/5 CBO representatives on 5/13, 1/5 on 6/12 and 7/18  2 ED attending physicians  Medical student coordinators | **Reflecting on impact and future directions:** Group discussion and walkthrough of Discharge Navigator IPV module, group discussion of ED residency curriculum development, summary slideshow | ED staff access to CBO resources regarding shelter bed availability  Challenges with continuously updating resources in ED  Access to resources in the ED for IPV survivors who may not disclose in the ED  Need for CBO input on ED residency curriculum |
