# Supplementary material for: Case Study and Qualitative Analysis of Emergency Department Community Advisory Council on Intimate Partner Violence
Source: West J Emerg Med. 2025 Dec 23;27(1):114–20. doi: 10.5811/westjem.47456 (PMC12815508; doi:10.5811/westjem.47456)
Supplement: Supplementary file 2 [file wjem-27-114-s002.docx]

APPENDIX 2: Interview Guide

[INTRODUCTION]
Hello, my name is [Interviewer’s name], and I’m conducting an evaluation of the [institution’s] Emergency Department Community Advisory Council focused on intimate partner violence. Your insight as a member is invaluable to us. This interview will take approximately 30 minutes. Would that be okay with you?

[CONSENT]
Before we begin, I want to assure you that your responses will be kept confidential and will be used for evaluation purposes only. Do you consent to participate in this interview?

[ICEBREAKER]
Could you start by telling me a bit about yourself, your organization and your role in the Community Advisory Council?

[COMMUNITY ADVISORY COUNCIL PROCESS]

1. Could you describe your overall experience participating in the council, starting with your thought process and reactions when you were invited to participate?
2. What do you believe are the strengths of the council’s process? What areas do you feel the council’s process could improve?
3. Prior to participating in the council, what experience, if any, did you have using human centered design? While participating in the council, were you aware that we were using human centered design principles?

[EMERGENCY DEPARTMENT AND CBO COLLABORATIONS]

1. How, if at all, has your organization collaborated with healthcare institutions in the past? Had you ever worked with [institution] or the [institution] ED before?
2. Prior to participating in the council, to what extent did you feel that the [institution] Emergency Department was actively engaged and partnered with community members? How, if at all, has participating in the council changed this?
3. How would you describe the level of community ownership in the council’s initiatives?
4. Can you describe your experience working with healthcare providers and Emergency Department staff via the council? How, if at all, do you feel that this model has impacted how your organization interfaces with the healthcare system?
5. Can you describe your experience collaborating with representatives from other organizations on the council? What are the strengths and downsides of this setting for collaboration between organizations?

[IMPACT & FEEDBACK]

1. What impact, if any, do you believe the council has had on addressing emergency medical care and Emergency Department-based support for survivors of intimate partner violence?
2. What suggestions do you have for improving the council’s effectiveness in addressing care and support for survivors in the Emergency Department?
3. How sustainable do you think the council’s initiatives are? What steps can be taken to make this collaboration sustainable for your organization?
4. What additional resources or support, if any, do you believe the council needs to be more effective?

[CLOSING]
Thank you for your time and insights. Your feedback is very valuable to us. Do you have any questions or is there anything else you would like to add?
